# Endoplasmic Reticulum Stress in the ****β****-Cell Pathogenesis of Type 2 Diabetes

**DOI:** 10.1155/2012/618396

**Published:** 2011-09-08

**Authors:** Sung Hoon Back, Sang-Wook Kang, Jaeseok Han, Hun-Taeg Chung

**Affiliations:** ^1^School of Biological Sciences, University of Ulsan, Daehak-ro, Nam-gu, Ulsan 680-749, Republic of Korea; ^2^Department of Medicine, Graduate School, University of Ulsan, Seoul, 138-736, Republic of Korea; ^3^Department of Biological Chemistry, University of Michigan Medical Center, Ann Arbor, MI 48109, USA

## Abstract

Type 2 diabetes is a complex metabolic disorder characterized by high blood glucose in the context of insulin resistance and relative insulin deficiency by *β*-cell failure. Even if the mechanisms underlying the pathogenesis of *β*-cell failure are still under investigation, recent increasing genetic, experimental, and clinical evidence indicate that hyperactivation of the unfolded protein response (UPR) to counteract metabolic stresses is closely related to *β*-cell dysfunction and apoptosis. Signaling pathways of the UPR are “a double-edged sword” that can promote adaptation or apoptosis depending on the nature of the ER stress condition. In this paper, we summarized our current understanding of the mechanisms and components related to ER stress in the *β*-cell pathogenesis of type 2 diabetes.

## 1. Introduction

Modern lifestyle, with overconsumption of energy-rich foods and reduced physical activity, has increased the rate of type 2 diabetes (T2D). T2D is a major cause of morbidity and mortality, decreasing both life quality and expectancy of affected individuals. Obesity is linked to insulin resistance and T2D [1]. In order to adapt to an increased metabolic load in obesity and insulin resistance, the normal pancreatic islets usually increase beta-cell mass through an increase in *β*-cell proliferation and neogenesis, as well as beta cell hypertrophy [2, 3] and enhancing *β*-cell function [4, 5]. However, failure of adaptation to the increased metabolic load results in a progressive decline in *β*-cell functions and cell death. As a consequence, individuals progress from normal glucose tolerance to impaired glucose tolerance and finally to established T2D [6, 7]. Accumulating evidence indicates that *β*-cell loss in T2D results from intertwined stress responses of gluco-/lipotoxicity, oxidative stress, and ER stress [6–14]. However, detailed molecular mechanisms underlying *β*-cell dysfunction and death remain to be clarified.

## 2. The Unfolded Protein Response in ***β*** Cells

The endoplasmic reticulum (ER) is a major subcellular compartment involved in calcium storage, lipid production, and protein biosynthesis in which a variety of extracellular signaling molecules and protein receptors critical for cellular homeostasis are properly folded, assembled, matured, and finally transported to their destination to function. These processes rely on the protein folding activity of chaperones densely populated in the ER [13]. However, folding activity can be overwhelmed with the amount of proteins imported into the ER under the instance of “ER stress”, during which unfolded proteins accumulate in the ER and trigger downstream signaling pathways, which is called the unfolded protein response (UPR) [15]. The UPR is triggered by three ER stress signaling transducers—PKR-like ER kinase (PERK, EIF2AK3), inositol requiring 1*α* (IRE1*α*), and activating transcription factor 6*α* (ATF6*α*)—on the ER membrane, resulting in attenuation of protein translation and transcriptional activation of UPR genes [15]. In addition, cells activate a pathway to dispose of misfolded proteins from the ER, termed “ER-associated degradation (ERAD)” [16]. Regulation of these processes from biosynthesis to degradation is required for protein homeostasis, and disruption in these processes can lead to terminal misfolding and/or aggregation of proteins in the ER. Then, terminally misfolded proteins which cannot be dealt by ERAD machineries need to be cleared from the ER by an additional process such as autophagy [17]. Thus, the adaptive pathways maintain cellular function and avoid apoptosis during ER stress. However, if ER stress is severe and chronic, UPR-mediated efforts to correct the protein folding defect fail, and the apoptotic pathway is preferentially activated over time [18, 19].

Increasing evidence indicates that ER stress is associated with a variety of diseases including diabetes, neurodegenerative disease, cancer, bipolar disease, liver disease, cardiac disease, muscle degeneration, autoimmune disease, and others [20, 21]. Several scientists have found evidence that T2D may be an example of an important human disease caused by ER stress [22, 23]. T2D occurs in patients who fail to compensate for insulin resistance by increasing insulin secretion. Therefore, pancreatic *β*-cell dysfunction and apoptosis are central to T2D pathogenesis. In this paper, we will discuss the complex ER stress responses responsible for *β*-cell protection as well as dysfunction and death during T2D.

### 2.1. Three Stress Response Pathways in the UPR

**Figure 1:**
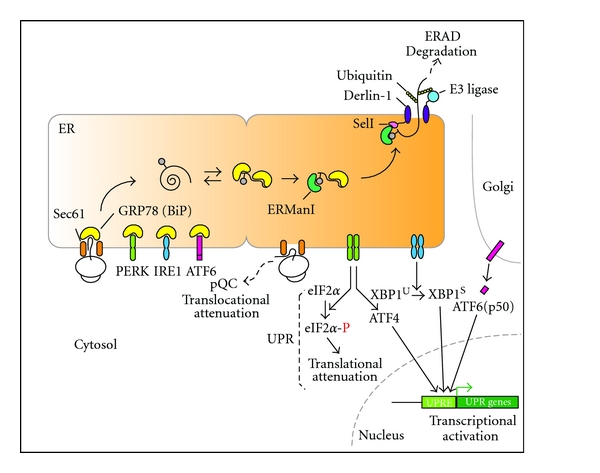
Activation of cellular responses during ER stress. In the resting state, newly synthesized polypeptides are cotranslationally translocated from the ribosome to the inside of the ER, in which GRP78 (BiP) plays two very important roles. First, GRP78 interacts and stabilizes polypeptides entering the ER and facilitates their proper folding, assembly, and maturation. Second, GRP78 interacts with PERK, IRE1*α*, and ATF6*α*, making them stay monomeric and functionally inactive on the membrane. However, these interactions are sensitive to protein folding status and can be easily disrupted by accumulation of misfolded proteins in the ER, resulting in activation of several pathways for protecting cells from accumulation of misfolded proteins: UPR, ERAD, and pQC. The pQC pathway is characterized by substrate-specific inhibition of protein translocation during ER stress, resulting in efficient degradation of mistranslocated proteins in the cytosol. This pathway is physiologically important in terms of controlling protein quantity in damaged ER. Following dissociation of GRP78 from ER stress sensors under ER stress, cells activate the UPR pathways to transfer signals to the nucleus and cytosol. PERK and IRE1*α* are autophosphorylated and modify their downstream signaling molecules, eIF2*α* phosphorylation, and *Xbp1* mRNA splicing, respectively. Phosphorylated eIF2*α* attenuates general protein translation in short. In addition, accumulation of phosphorylated eIF2*α* induces ATF4 expression. Together with ATF4, spliced XBP1 and cytosolic fragments of ATF6*α* (p50) transcriptionally activate various UPR genes involved in either adaptation or apoptosis during ER stress.

Cells have evolved an intertwined three cellular pathway termed “the unfolded protein response” to prevent accumulation of misfolded proteins in the ER lumen. ER stress such as misfolded protein accumulation is sensed by the luminal domains of three ER transmembrane proteins: PERK, IRE1*α* and ATF6*α*. Then activated stress sensors initiate the complex signaling pathways (Figure 1) [15].

#### 2.1.1. PERK Pathway

During ER stress, PERK is dissociated from GRP78 (BiP), an abundant ER chaperone, then multimerizes and autophosphorylates [24]. Activation of PERK leads to phosphorylation of the alpha subunit of eukaryotic initiation factor 2 (eIF2*α*), which is an early response required for the attenuation of global protein translation in response to ER stress aimed to prevent further overload of the nascent polypeptides to be folded in the ER lumen [15, 25, 26]. PERK, on the other hand, induces efficient translation of several specific transcripts (such as cationic amino acid transporter 1 (cat-1), growth arrest, and DNA damage 34 (GADD34), ATF5, and ATF4) even under the condition of significant eIF2*α* phosphorylation [27–29]. Among them, translational increase of ATF4 induces expression of several genes involved in ER protein folding, ERAD, amino acid biosynthesis and transport function, and antioxidative stress response. Thus, translational inhibition to general mRNA transcripts but translational activation to specific mRNA transcripts by PERK is an important component of the UPR-mediated adaptation pathways to ER stress [29, 30]. Therefore, PERK activity and eIF2*α* phosphorylation are particularly important to maintain function of pancreatic *β*-cells, which continuously synthesize and secrete large amounts of insulin molecules according to physiological fluctuation of blood glucose [29, 31]. Several studies discussed below have shown that disruption of their activities increases susceptibility of *β* cells to death and induces *β*-cell failure associated with insulin resistance in T2D [23, 32, 33]. In contrast, PERK also induces expression of CHOP through transcriptional activation by ATF4 [34], an important proapoptotic gene of ER stress-mediated *β*-cell death [35, 36]. Therefore, it is now believed that the PERK-mediated signaling pathway may behave like a binary switch determining life and death of *β* cell depending on the nature of ER stress condition.

#### 2.1.2. IRE1*α* Pathway

The luminal domain of PERK is functionally interchangeable in transmitting ER stress signal with IRE1*α*, another ER sensor, from which GRP78 is released upon exposure to ER stress [24]. IRE1*α*, like PERK, is autophosphorylated and dimerized by its cytoplasmic kinase domain as misfolded proteins in the ER accumulate, leading to activation of the C-terminal ribonuclease domain and specific cleavage of mRNA encoding a basic leucine zipper containing transcription factor X-box-binding protein (XBP1) [37–39]. The spliced *Xbp1* mRNA encodes a strong transcription factor (XBP1s) for many UPR genes important in protein folding, trafficking, secretion, and ER-associated degradation [40–42]. Therefore, the transcriptional function of XBP1s is important for many professional secretory cells, particularly, *β* cells [42, 43]. Thus, the IRE1*α*-XBP1 pathway contributes to restoring ER homeostasis to meet protein folding demand and protein transport [44]. Beside homeostatic function, IRE1*α*, under chronic ER stress, also activates proapoptotic c-Jun N-terminal kinase (JNK) signaling pathway and interacts with members of the B-cell lymphoma 2 (BCL2) family, causing cellular dysfunction and apoptosis [15, 45]. Moreover, it has been shown more recently that endonuclease activity of IRE1*α* cleaves ER-localized mRNAs, including proinsulin mRNA, resulting in *β*-cell dysfunction and apoptosis [46, 47].

#### 2.1.3. ATF6 Pathway

The stress sensors mediating the third UPR pathway activating transcription factor *α* (ATF6*α*) and ATF6*β*, a structural homologue of ATF6*α* [48], are also associated with GRP78 and retained in the ER membrane. During ER stress, both proteins released from GRP78 traffic to the Golgi apparatus [49, 50] from which their active cytosolic fragments (p50ATF6*α* and p60ATF6*β*) are generated by S1P and S2P protease and migrate into the nucleus [51]. Although the activation mode of ATF6*β* during ER stress seems the same to ATF6*α* and biochemical studies to ATF6*β* suggest it has similar biological functions to ATF6*α*, analysis of mouse embryonic fibroblasts (MEFs) deficient in ATF6*α* or ATF6*β* revealed that ATF6*α* but not ATF6*β* is responsible for transcriptional induction of ER chaperones including GRP78 and that p50ATF6*α* heterodimerized with XBP1s are capable of binding both ER stress response element (ERSE) and UPR elements (UPRE) conserved in the promoters of UPR genes, resulting in significant activation of genes to restore proper ER function, protein folding, and ERAD [52, 53]. However, double knockout of ATF6*α* and ATF6*β* caused embryonic lethality whereas ATF6*α*-or ATF6*β*-deficient mice are dispensable for embryonic and postnatal development, respectively, these results suggest that ATF6*α* and ATF6*β* possess at least an overlapping function which is essential for mouse development [52, 53]. Although ATF6*α*-null murine model did not show pancreatic *β*-cell demise from functional deficiency of ATF6*α*  [52, 53], hyperactivation of ATF6*α* decreases insulin gene expression via upregulation of the orphan nuclear receptor small heterodimer partner (SHP; NR0B2) which has been shown to play a role in *β*-cell dysfunction [54].

## 3. ERAD and Non-ERAD Mechanisms

The UPR involves three distinct mechanisms, namely transcriptional induction of ER-resident chaperones to facilitate protein folding, translational attenuation to decrease the demand of protein folding, and ERAD to degrade the unfolded proteins accumulated in the ER lumen. Emerging data now indicate that the function of the UPR restoring ER homeostasis is facilitated by both ERAD and non-ERAD (such as autophagy and preemptive quality control) mechanisms to remove aggregated proteins from the ER and reduce new substrates during stress. Thus, efficient removal of misfolded proteins is essential to protect cells from ER stress. Here, we will review the protein degradation pathways associated with the ER.

### 3.1. ER-Associated Degradation (ERAD) Pathway

Eukaryotic cells have protein quality and quantity control systems aimed to dispose of misfolded proteins from the ER [55]. Consequently, chaperones in the ER distinguish physical differences between properly folded and unfolded proteins in the ER [56, 57]. Hsp70-type (such as GRP78) and glycan-dependent chaperones (such as calreticulin and calnexin) bind unfolded proteins and contribute to maintain solubility of substrates, leading to remodeling of proteins that have incorrect conformation [57–59]. Efficient removal of misfolded proteins by the ERAD pathway seems to be very specific because it directly deals with specific substrates and, apparently, is essential to protect cells from ER stress and restore proper ER function. Several distinct steps complete this pathway. First, although the mechanism selecting substrates is still under investigation, when cells recognize terminally misfolded proteins unable to acquire their native structures [60], ER-mannosidase I (ERManI) and mannosidase-like proteins (EDEMs) flag a target glycoprotein for degradation and activate the degradation pathway thereafter [61–63]. Second, substrates are retrotranslocated across the ER membrane via multicomplex channels, including Sec61, Derlin-1, or E3 ubiquitin ligase family members [64–66]. Third, target proteins are ubiquitylated by an E3 ligase. Finally, target proteins are then removed from the ER membrane and transported to the proteasome for degradation. Although the importance of the ERAD mechanism in pancreatic *β*-cells has not been studied extensively, recent studies suggest that defective protein degradation by reduction of ubiquitin carboxyl-terminal hydrolase L1 (UCH-L1) activity can compromise viability in *β* cells in T2D. Downregulation of UCH-L1 expression and activity in *β* cells induces ER stress and apoptosis [67]. In addition, E3 ubiquitin ligase HRD1 may have a protective role as an ubiquitin ligase for ATF6*α* [68], which inhibits hyperactivation of ATF6*α* in the islets of WFS1-deficient mice.

### 3.2. Autophagy

While ERAD controls the degradation of smaller units of unfolded and misfolded proteins, larger aggregates and long-lived proteins are detoxified via degradation in the lysosome, a process called autophagy [69]. Autophagy was originally identified as a dynamic process for degradation of cytosolic organelles [70]. Now it has also been addressed as an additional degradation pathway for proteins strongly linked to the UPR pathway [69]. For example, the phosphorylation of eIF2*α* is also required for the induction of autophagy [71]. Therefore, ER stress stimulates autophagy as an adaptive response to clean up terminally misfolded proteins from the ER. 

### 3.3. Preemptive Quality Control (pQC)

In addition to typical quality control pathway in mammals such as ERAD, a new degradation pathway for secretory proteins has recently been discovered. During acute ER stress, some secretory and membrane proteins are rerouted in a signal sequence-selective manner from its normal fate of being translocated into the ER to a pathway of proteasome-mediated degradation. Their cotranslational rerouting to the cytosol for degradation reduces the burden of misfolded substrates entering the ER, termed this process pre-emptive quality control (pQC) [72] For example, prion protein (PrP) is mistranslocated and rerouted to the cytosol for immediate degradation by the proteasome during ER stress. This process is largely regulated by the specific signal sequence of proteins [72, 73].

**Figure 2:**
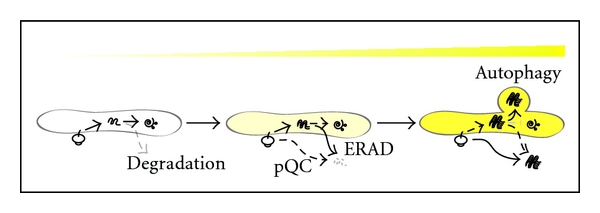
Potential activation mechanisms for disposing misfolded proteins from the ER. Misfolded proteins in the ER can be disposed of by serial activation of ERAD, pQC and autophagy according to the degree of protein misfolding and aggregation. In general, a small amount of protein is spontaneously misfolded and efficiently degraded in the ER even in the resting state. Under ER stress, accumulation of misfolded proteins in the ER activates pQC to reduce the burden of proteins in damaged ER as well as the ERAD pathway to dispose of misfolded proteins from the ER. However, under prolonged ER stress, ERAD does not efficiently dispose of and degrade protein aggregates from the ER, resulting in activation of an alternative way to clean them up from the ER, called “autophagy”. Activation of these pathways is aimed at increasing ER capacity for protecting cells from misfolded proteins.

Efficient UPR pathway activated at the early stage of ER stress readily remodel misfolded proteins and restore proper ER function. As ER stress is excessive and prolonged, terminally misfolded proteins are disposed of from the ER by the ERAD pathway. At the same time, the pQC pathway reroutes misfolded proteins from the ER to the cytosol for degradation, leading to a reduction in the burden of misfolded substrates entering the ER. Therefore, when the UPR and/or ERAD pathways are compromised, the pQC pathway is apparently beneficial for cells under ER stress (Figure 1). Furthermore, to remove large aggregations, cells activate autophagy by which a large portion of aggregations can be transported directly to lysosomes for degradation without passing through the Golgi (Figure 2). However, terminally misfolded proteins often accumulate and aggregate in the ER. When the previously mentioned protein degradation mechanisms are not functionally efficient in dealing with the increasing amount of substrates, cells fail to be rescued from accumulation of misfolded proteins in the ER and cytosol. This may activate several ER stress-mediated pro-apoptotic pathways resulting in the death of stressed cells [74].

## 4. Roles of UPR-Related Genes in ***β*** Cells

### 4.1. PERK/eIF2*α* Pathway

A rare human autosomal recessive genetic disorder, the Wolcott-Rallison syndrome, is characterized by early infancy diabetes, multiple epiphyseal dysplasia, and growth retardation [75, 76]. These patients have endocrine and exocrine insufficiency and pancreatic atrophy with reduced number of *β*-cells [77, 78]. This syndrome was found to be associated with mutations in the *Perk* gene. In addition to that, linkage between diabetes and *Perk* gene was reported in Scandinavian families [79] and South Indian populations [80]. 

In addition to human studies, investigations using *PERK^−/−^* mice and mice (*Ser51Ala*) with mutation in the phosphorylation site of eIF2*α* showed a potential relationship between ER stress and *β*-cell function [29, 30, 81]. Pancreatic *β* cells developed normally in whole body *Perk*-null mice but showed a diabetic phenotype soon after birth mainly due to *β*-cell death [30, 81, 82]. In these studies, ER distention was observed in pancreatic *β* cells. In addition, there was more proinsulin production in *Perk^−/−^* *β* cells challenged by high glucose, causing them to experience higher translational loads and higher levels of ER stress than *wild type*. These results suggested that ER overload and unresolved ER stress may cause *β*-cell death in those mice. Conditional deletion of *Perk* at different developmental stages showed that PERK expression in *β*-cells is not required postnatally in adult mice to maintain glucose homeostasis, whereas its expression is a prerequisite for fetal/neonatal *β* cell proliferation and differentiation [82]. 

Similar to *Perk^−/−^* mice, *eIF2*α** homozygous mutant mice showed deficiency in pancreatic *β* cells in the embryonic stage, and they died within 18 hr after birth [29]. Moreover, the *β* cells of late embryonic stage in the mutant mice have reduced insulin contents and show severe distension of the ER [31, 33]. The results suggest that function of eIF2*α* phosphorylation may be required for *β*-cell differentiation and proliferation, along with embryonic *β* cells of *Perk^−/−^* mice. However, recent results showed that eIF2*α* phosphorylation but not PERK expression in *β* cells is required at the adult stage to maintain *β*-cell functions and glucose homeostasis. The absence of eIF2*α* phosphorylation in *β* cells caused defective intracellular trafficking of ER cargo proteins, increased oxidative damage, and reduced expression of stress response and *β*-cell-specific genes and apoptosis due to heightened and unregulated proinsulin translation [33]. Since Ser51Ala homozygous mutation in *eIF2*α** (*A/A*) does not allow any compensatory phosphorylation by other eIF2*α* kinases (such as heme-regulated inhibitor kinase (HRI, EIF2AK1), general control of nitrogen metabolism kinase 2 (GCN2, EIF2AK4), and dsRNA-activated protein kinase (PKR, EIF2AK2)) [83] in adult *β* cells in response to physiological stimuli, whereas other eIF2*α* kinase in PERK-deficient adult *β* cells might compensate the requirements for eIF2*α* phosphorylation. This characteristic might contribute to phenotypic discrepancies between *Perk^−/−^* and *A/A* mice. The heterozygous mutant (S/A) mice did not spontaneously develop diabetes; however, on a 45% high fat diet, these mice showed glucose intolerance and insulin secretion defect in *β* cells [31]. These results suggested that translational regulation through eIF2*α* phosphorylation is required to maintain functional integrity of the ER. This hypothesis was further demonstrated by a study using mice with conditional A/A mutation in *β* cells [33]. The *A/A* mice were rescued by transgenic expression of wild-type eIF2*α* cDNA, which could be specifically deleted in *β* cells by tamoxifen-inducible Cre recombinase. As early as 3 weeks after deletion of wild-type eIF2*α* cDNA, *β* cells showed significantly distended ER and swollen mitochondria [33].

### 4.2. WFS1

Wolfram syndrome (WFS) is a rare autosomal recessive neurodegenerative disorder characterized by early onset diabetes, optic atrophy, and hearing impairment [84]. This syndrome is genetically linked with mutations in the *Wfs1* gene that encodes the protein wolframin [85, 86]. As in human cases, mice deficient in the *Wfs1* gene developed glucose intolerance and overt diabetes due to insufficient insulin secretion. Pancreatic *β* cells in mutant mice experienced ER stress shown by phosphorylation of eIF2*α* and spliced form of XBP1 [87–89]. Recent study suggests that WFS1 may affect maturation of plasma membrane proteins or stability of ER membrane proteins. Deficiency of WFS1 in *β* cells destabilizes Na^+^/K^+^ ATPase *β*1 subunit and E3 ubiquitin ligase HRD1 [68, 90]. Thus, wolframin may be involved in the ER folding and assembly of subunits of oligomeric proteins. In addition, Wolframin may suppress ATF6*α* hyperactivation in *β* cells by stabilizing HRD1, which brings ATF6*α* to the proteasome. Therefore, WFS may have a role as a negative regulator of chronic or unresolvable ER stress.

### 4.3. P58^IPK^

Although p58^IPK^ (IPK, inhibitor of protein kinase) was first known to be an inhibitor of the PKR, its function has been shown to inhibit another eIF2*α* kinase, PERK. However, recent evidence suggests that p58^IPK^ serves as a cochaperone in the ER lumen for the Hsp70 family member BiP [91]. Mice lacking the p58^IPK^ gene showed gradual onset of glucosuria and hyperglycemia mainly due to apoptosis of pancreatic *β* cells [92]. In addition, *p58^IPK^* deletion in *Akita* mice (carrying a C96Y mutation in the *Ins2 *gene) exacerbate the diabetic phenotype [93]. These results indicate that chaperoning ability in the ER is important to preserve ER function in *β* cells.

### 4.4. ATF6*α*

ATF6*α* was also found to be associated with *β*-cell function in genetic studies. In a study of Pima Indians, a native American population with a high prevalence of type II diabetes, [94] they found an association of variants in ATF6 with T2D [95]. In another study conducted in a Dutch cohort, they also found that the majority of single nucleotide polymorphisms (SNPs) in *Aft6* allele were significantly associated with impaired fasting glucose, impaired glucose tolerance, and T2D [96]. Furthermore, associated variants differed from those identified in the Pima Indians. Since ATF6*α* is important for protective cell response to accumulation of unfolded and misfolded proteins in the ER, disturbances of this process might contribute to *β*-cell apoptosis. However, there is no direct evidence of pancreatic *β*-cell demise in the ATF6*α* null murine model.

## 5. Causes of ER Stress in ***β*** Cell

Increasing evidence indicates that ER stress is one of the main causes of *β*-cell dysfunction and death [6–14]. However, it is not clear what is the main cause of ER stress in *β* cells, and which subpathway of UPR is responsible for this process. In this section, we will describe the potential sources and mechanisms for ER stress-mediated *β*-cell demise. 

### 5.1. Lipotoxicity-Mediated ER Stress

Hyperlipidemia (elevated serum lipid levels) also results from sustained insulin resistance. It is thought that chronically elevated levels of circulating free fatty acids (FFAs) are putative mediators of progressive *β*-cell dysfunction and death in T2D [97]. When FFA levels are elevated twofold above the basal upon lipid infusion, obese nondiabetic individuals showed a significant reduction in glucose-stimulated insulin release [98]. It has recently been shown that a saturated long-chain FFA palmitate induces ER stress in both clonal and primary murine and human *β* cells, whereas unsaturated long-chain FFAs do or do not induce ER stress to a lesser content [10, 11, 99, 100]. Palmitate preferentially activates both PERK and IRE1*α* pathways [101–103]. However, it is uncertain whether palmitate can specifically activate the ATF6*α* branch because both palmitate and oleate induce total *Xbp1* mRNA, and the expression of the known ATF6*α* target gene *Hspa5 *(*Grp78*) is controversial [102, 104]. How saturated FFAs activate the unfolded protein response has not been answered. Several recent studies indicate that palmitate triggers ER stress in *β* cells through perturbation in ER Ca^2+^ handling. Calcium-specific dye assays revealed palmitate depletes ER Ca^2+^ and slows ER Ca^2+^ uptake in *β* cells [104]. Although palmitate-mediated ER Ca^2+^ depletion increased misfolded protein accumulation as the mechanism of the sarco-/endoplasmic reticulum Ca^2+^-ATPase (SERCA) inhibitors thapsigargin and cyclopiazonic acid, two commonly known synthetic ER stressors, the detailed molecular mechanism of ER Ca^2+^ depletion by palmitate is not well known. Other reports indicated palmitate rapidly increases the saturated lipid content of the ER, leading to compromised ER morphology and integrity and thereby may directly or indirectly induce ER stress [100, 105]. For example, impairment of lipid content control of the ER by palmitate hampers ER-to-Golgi protein trafficking of vesicular stomatitis virus G protein (VSVG), contributing to the unfolded protein response through accumulation of misfolded proteins in the ER lumen [106]. Interestingly, increased fatty acid desaturation by stearoyl coenzyme A desaturase 1 (SCD1) reduced palmitate-induced cell death in MIN6 *β* cells and human embryonic kidney (HEK) cells [107]. In contrast, knockdown of SCD in INS-1 *β* cells decreased desaturation of palmitate to monounsaturated fatty acid (MUFA), lowered fatty acid partitioning into complex neutral lipids, and augmented palmitate-induced ER stress and apoptosis [108]. Furthermore, the importance of lipid content control of the ER by SCD in *β* cells was further manifested by studies of diabetic murine models. First, loss of SCD1 worsens diabetes in leptin-deficient obese mice [109]. Second, SCD1 and SCD2 mRNA expression were shown to be induced in islets from prediabetic hyperinsulinemic Zucker diabetic fatty (ZDF) rats, whereas several fatty acid desaturases including SCD1 mRNA levels were markedly reduced in diabetic ZDF rat islets [108].

### 5.2. Misfolded Protein-Mediated ER Stress

Since proinsulin represents up to 20% of the total mRNA and 30–50% of the total protein synthesis in the *β* cell [110–112], misfolded mutant insulin proteins might be a potent cause of ER stress. The Akita mutant in both mouse and human, which carries a cysteine 96 to tyrosine substitution in the *Ins2* gene, showed hyperglycemia and a reduced *β* cell mass [35]. This missense mutation disrupts a disulfide bond formation of mature insulin causing incorrect folding of proinsulin in the ER. Accumulation of the mutant insulin induced cell death mainly through ER stress evidenced by upregulated expression of ER stress marker genes such as BiP, spliced XBP1s, activated ATF6*α*, and CHOP in the Akita mutant islets and *β*-cell lines [113–115]. In heterozygous, but not homozygous, Akita's mutant mice, the homozygous disruption of CHOP delayed diabetes development suggested that *β*-cell death is partially CHOP dependent [93]. 

Similar to Akita diabetes due to accumulation of misfolded proinsulin, ER accumulation of islet amyloid polypeptide (IAPP, amylin) oligomers may contribute to *β*-cell loss in T2D [12]. Human IAPP is an 89 amino acid protein that undergoes processing to a 37-amino acid amyloidogenic peptide coexpressed and cosecreted with insulin by *β* cells. A study showed that islet amyloid is present at autopsy in over than 90% of patients with T2D [8]. Moreover, increased expression of ER stress marker genes such as BiP and CHOP was observed in islets of human T2D patients [22]. The IAPP-driven ER stress theory was exemplified by animal studies overexpressing human IAPP in *β* cells. Human IAPP but not murine IAPP forms toxic oligomers and triggers ER stress-induced apoptosis in *β* cells of both rat and mouse murine models [12]. Therefore, intracellular deposit of human IAPP toxic oligomer could be a link between ER stress and *β*-cell death in human T2D.

### 5.3. High Glucose-Mediated ER Stress

Insulin resistance in T2D causes blood glucose levels to remain high [116]. In a high blood glucose state, called hyperglycemia, the *β*-cell increases its metabolic activity, which eventually leads to cellular stress. This in turn may further impair *β*-cell function and survival, a process called glucotoxicity [117]. It has been shown that glucotoxicity is mediated at least in part by excess generation of reactive oxygen species (ROS) [117, 118]. When excess glucose is available to the *β* cell, excessive ROS can be generated in *β* cells by several biochemical pathways including mitochondrial oxidative phosphorylation, ER oxidative folding pathway, and other alternative metabolism pathways; overflowed glucose is shunted (such as glucosamine and hexosamine metabolism and sorbitol metabolism) [117, 118]. Elevated ROS perturbs insulin synthesis and secretion by decreasing the expression and activity of key transcription factors such as PDX-1 and MafA, which regulate proinsulin genes and other multiple genes involved in *β*-cell differentiation, proliferation, and survival [119]. 

Accumulating evidence suggests that protein folding in the ER and production of ROS are closely linked events [120]. In several reports, prolonged UPR activation leads to the accumulation of ROS via two sources: the UPR-regulated oxidative protein folding machinery in the ER and oxidative phosphorylation in mitochondria [118, 121]. In the ER lumen, oxidative protein folding is conducted by protein disulfide isomerase (PDI) and a family of ER oxidoreductases 1 (ERO1) that catalyze disulfide bond formation in folding proteins. In this reaction, an oxidant flavin adenine dinucleotide (FAD)-bound ERO1 oxidizes PDI, which then subsequently oxidizes folding proteins directly. FAD-bound ERO1 then passes two electrons to molecular oxygen, perhaps resulting in the production of ROS such as hydrogen peroxide or peroxide [120]. Overexpression of a misfolded protein CPY (yeast vacuolar protein carboxypeptidase Y) activates the UPR, causes oxidative stress, and induces apoptosis. However, removal of all cysteine residues in CPY reduced oxidative stress and cell death [122]. Therefore, oxidative protein folding in the ER can be a source of ROS generation. It is known that ER stress increase leakage of Ca^2+^ from the ER lumen through mainly the inositol-1,4,5-trisphosphate receptor (IP_3_R) [21]. Recent studies suggest that ER Ca^2+^ leakage may occur by oxidation-induced activation of the Ca^2+^ release channel IP_3_R during ER stress and oxidative stress [123]. Increases in cytosolic Ca^2+^ can stimulate mitochondrial ROS production through multiple mechanisms [124]. Introduced by Ca^2+^ uniporter or mitochondrial ryanodine receptor, Ca^2+^ stimulates the TCA cycle and nitric oxide synthase (NOS), which subsequently generates nitric oxide. Nitric oxide and Ca^2+^ inhibits respiration complex I, III, or IV, which enhance ROS generation. High levels of ROS generation within the mitochondria then further increase Ca^2+^ release from the ER. In turn, Ca^2+^ also dissociates cytochrome *c *from the inner membrane cardiolipin, which triggers permeability transition pore (PTP) opening and cytochrome *c *release across the outer membrane. Now the vicious cycle of ER Ca^2+^ release and mitochondrial ROS production activates cytochrome-*c*-mediated apoptosis. The ER of diabetic pancreatic *β* cells synthesizing great quantities of proinsulin to maintain normoglycemia can be an important site of ROS production because correct folding of proinsulin absolutely depends on the formation of disulfide bond by oxidative protein folding. This theory is partially supported by recent reports that PERK and eIF2*α* phosphorylation-deficient *β* cells having reduced UPR responses showed increased proinsulin synthesis due to losing of translational control; thereby, large accumulation of proinsulin in the ER attributed to accumulated ROS in *β* cells possibly by both ER oxidative protein folding machinery and Ca^2+^ mediated-mitochondria activation [33, 82]. Thus, excess ROS and the mitochondrial cell death pathway may induce *β*-cell death. Therefore, the finding that increased proinsulin synthesis causes oxidative damage in *β* cells may reflect events in *β*-cell failure associated with insulin resistance in T2D [74].

Under high glucose conditions, it is possible that increased proinsulin biosynthesis may overwhelm the ER protein folding capacity leading to UPR activation. Chronic exposure (more than 24 hrs) of *β* cells to high-glucose caused hyperactivation of IRE1*α* showing *Xbp1* mRNA splicing, whereas acute expose (1–3 hrs) to high glucose activated IRE1*α* without *Xbp1* mRNA splicing [47, 118, 125]. In the chronic high-glucose state, activated IRE1*α* degrades proinsulin mRNA contributing to the reduction of proinsulin biosynthesis [47, 125]. Moreover, recent reports suggest that IRE1*α*'s RNase causes endonucleolytic decay of many ER's localized mRNAs, including those encoding chaperones, thereby culminating in cellular dysfunction and death of several mammalian cells including *β* cells [46, 47, 125]. However, the studies also revealed that activated IRE1*α* at the physiological level may have a beneficial effect aiding in the enhancement of proinsulin biosynthesis in pancreatic *β* cells with the induction of a subset of downstream genes of IRE1*α*  [47]. Thus, depending on the forms of ER stress, *β* cells may generate binary signaling of life and death through IRE1*α*. 

It is well established that high blood sugar amplifies FFA-mediated toxicity in *β* cells [117, 118]. Although why glucose exacerbates *β*-cell lipotoxicity is not well known, a recent report suggests that chronic hyperglycemia may amplify fatty acid-induced ER stress in *β* cells [101]. The study showed that high glucose amplifies palmitate-mediated stimulation of the IRE1*α* and PERK pathways. Glucose stimulates the mammalian target of rapamycin complex 1 (mTORC1), an important nutrient sensor involved in the regulation of cellular stress, and the activated mTORC1 mediates amplification of fatty acid's lipotoxicity by increasing IRE1*α* protein levels and activating the JNK pathway, leading to increased *β*-cell apoptosis.

## 6. Concluding Remarks

Increasing evidence indicates that hyperactivation of the UPR indispensible for ER homeostasis has a role in *β* cell dysfunction and death during the progression of T2D and genetic forms of diabetes. Therefore, it is currently believed that ER stress-related diseases including T2D occur from adaptation to apoptosis of stressed cells. The complete understanding of this molecular mechanism responsible for life and death will shed light on future T2D prevention and treatment.
